# Comparative Analysis of Adult Patients With Idiopathic Pulmonary Hemosiderosis and Lane-Hamilton Syndrome: A Systematic Review of the Literature in the Period 1971-2022

**DOI:** 10.7759/cureus.23482

**Published:** 2022-03-25

**Authors:** Biplab K Saha, Praveen Datar, Alexis Aiman, Alyssa Bonnier, Santu Saha, Nils T Milman

**Affiliations:** 1 Pulmonary and Critical Care Medicine, Ozarks Medical Center, West Plains, USA; 2 Internal Medicine, New York Institute of Technology College of Osteopathic Medicine at Arkansas State University, Jonesboro, USA; 3 Critical Care, Goldfarb School of Nursing at Barnes Jewish College, Saint Louis, USA; 4 Internal Medicine, Saha Clinic, Narail, BGD; 5 Pulmonary and Critical Care, University College Zealand, Næstved, DNK

**Keywords:** coexisting, lane hamilton syndrome, celiac disease, adult, idiopathic pulmonary hemosiderosis

## Abstract

Idiopathic pulmonary hemosiderosis (IPH) causes diffuse alveolar hemorrhage (DAH) by a yet unknown mechanism. The coexistence of IPH and celiac disease (CD), also known as Lane-Hamilton syndrome (LHS), has been reported in both pediatric and adult patients. The objective of this study was to compare demographics, clinical and radiologic findings, treatment, and outcomes between adult patients with IPH and LHS.

This is a systematic review of the literature. Multiple databases were searched using appropriate formulas to identify relevant articles. A total of 60 studies reporting 65 patients were included in the review. Forty-nine of these patients had IPH and 16 had LHS. The prevalence of anti-CD antibodies among tested patients was 13/22 (59%). The symptom onset and diagnosis of IPH occurred earlier in patients with LHS. The median delay in diagnosis was the same between the two groups (52 weeks). The classic triad was more likely to be present in patients with LHS. Only 20% of patients in the LHS cohort had any significant gastrointestinal (GI) symptoms at the time of IPH diagnosis. A gluten-free diet alone was effective in the majority of patients. Fewer patients in the LHS cohort received systemic corticosteroid than the IPH cohort. The recurrence and mortality in patients with LHS appear to be less than in the IPH cohort. The prevalence of CD is 25% in adult patients with IPH. Patients with LHS may have a milder course than patients without CD. Serologic testing for CD should be performed in all patients diagnosed with IPH.

## Introduction and background

Idiopathic pulmonary hemosiderosis (IPH) is a rare disease that only affects the lungs. IPH causes diffuse alveolar hemorrhage (DAH) by an unknown mechanism and is often recurrent. An appropriate diagnosis of IPH requires careful exclusion of all competing diagnoses and histopathologic proof of ‘bland pulmonary hemorrhage’[1-4]. The histopathologic hallmark of IPH includes evidence of recent and/or prior alveolar hemorrhage, extracellular or intracellular deposition of hemosiderin in the alveolar macrophages, type 2 pneumocyte hyperplasia, and the absence of necrosis, vasculitis, granulomatosis, immunocomplex deposition, or inflammatory cellular infiltration of the pulmonary interstitium [5-6]. The repeated episodes of pulmonary hemorrhage result in local iron overload, causing pulmonary hemosiderosis. Additionally, chronic oxidative damage by heme and free radicles may result in pulmonary fibrosis, chronic respiratory impairment, or even end-stage lung disease [7-10].

Celiac disease (CD) is a common autoimmune disease with well-known intestinal and extraintestinal manifestations. The coexistence of IPH and CD, also known as Lane-Hamilton syndrome (LHS), was first reported by Drs. Lane and Hamilton more than 50 years ago [11]. Whether this is a mere coincidence, or an underlying unifying pathobiology exists, is unknown. It is also unclear if there is any difference regarding demographics, clinical presentations, and prognosis between patients with IPH and LHS. Anecdotally, some authors have reported positive outcomes with a gluten-free diet (GFD) without immunosuppression in patients with LHS, but there have been no prospective or retrospective studies to address these questions. In this manuscript, we have performed a thorough review of the literature to elucidate the similarities and differences between adult patients with IPH and LHS.

## Review

Materials and methods

This is a systematic review of the existing literature. The review was performed and reported according to the Preferred Reporting Items for Systematic Review and Meta-Analyses (PRISMA) guidelines [12].

Creation of Patient Cohorts

The Medline, PubMed, and Embase databases were searched using the following formula:

‘Idiopathic pulmonary hemosiderosis OR IPH AND adults’; ‘idiopathic pulmonary hemosiderosis OR IPH AND celiac disease’; ‘idiopathic pulmonary hemosiderosis OR IPH AND celiac disease’; and ‘idiopathic pulmonary hemosiderosis OR IPH AND celiac disease AND adult.’ Two independent investigators (BKS and AB) abstracted each identified study and compared their results. The reviewers were blinded to each other's assessment. Any disagreement between the researchers was resolved by discussion and input from a third investigator, SS. After the citations were identified, we removed duplicate citations. We then screened the abstract of each citation for applicability to our review. Citations that were deemed unrelated to our research after an independent evaluation of the abstract by the reviewers were excluded. The full texts of the remaining citations were then reviewed, along with a careful examination of the bibliography of the published articles. The identified patients were then divided into two cohorts: 1) adult patients with IPH without CD, cohort A, and 2) Adult patients with LHS, cohort B. Strict inclusion and exclusion criteria were followed while identifying patients with IPH from the literature.

Study Objectives

The primary objectives of the study were to compare the following parameters between adult IPH patients with (LHS) or without CD: 1) demographics, 2) age at symptom onset, 3) age at diagnosis, 4) duration between symptom onset and diagnosis of IPH, 5) clinical presentation, 6) radiologic abnormalities, 7) treatment, and 8) prognosis and survival.

The secondary objectives were to report histopathologic findings from small bowel biopsy and non-pulmonary organ involvements in patients with LHS.

Inclusion Criteria

Strict inclusion criteria were followed during the selection of appropriate studies. Articles fulfilling the following criteria were included in this review: 1) prospective or retrospective studies that reported the occurrence of IPH in patients age 18 and above; 2) case reports or case series that reported patients with LHS; 3) cases where the diagnosis of IPH was made with consistent clinical and radiologic findings and demonstration of hemosiderin-laden macrophages (HLM) from respiratory tract samples, obtained either by bronchoscopy or spontaneous expectoration (sputum), and/or lung biopsy consistent with IPH; 4) articles published in the English language in peer-reviewed journals between January 1, 1971, and February 21, 2022.

Exclusion Criteria

The exclusion criteria were as follows: 1) articles where the patient was diagnosed with IPH as a child, even if the patient was reported as an adult; 2) articles that reported autoantibodies, demonstrated signs and symptoms of vasculitis, and had lung or kidney biopsies consistent with vasculitis during the initial diagnosis of IPH (these patients were considered to be suffering from primary vasculitides); 3) patients with bland pulmonary hemorrhage with clinical and laboratory findings diagnostic of a connective tissue disease during initial diagnosis (pulmonary hemorrhage in these patients were determined to be secondary to the rheumatologic disease and the patient was not labeled with IPH), however, if a patient with biopsy-proven IPH developed a connective tissue disease years later, the patient was included, as the causation could not be proven definitively; 4) pediatric patients with IPH and autoantibodies; these have been reported in a recent paper [13]; 5) patients reported as meeting abstracts; 6) patients reported in a non-English language.

Data Items

Included studies were coded, and the extracted data from the studies were then tabulated in a standardized Excel sheet (Microsoft Corporation, Redmond, WA). The following data were gathered from all full-text articles: study design, year of reporting, country of the study, patient demographics, presenting symptoms, delay in the diagnosis of IPH, types of tested autoantibodies, type of positive autoantibodies, the temporal relationship of autoantibody determination with the diagnosis of IPH, diagnostic modality, lung biopsy results when available, treatment, and clinical outcome of the disease. Additional data were collected relevant to CD for patients with LHS. These included small intestinal biopsy and evidence of other organ involvement.

Study Risk and Bias Assessment:

The risk of bias was not assessed, as the identified papers were primarily case reports and case series.

Statistical Analysis

Descriptive and inferential statistical analyses were performed using the IBM SPSS statistical software package, version 28 (IBM Corp., Armonk, NY). The normality of distribution was assessed by the Shapiro-Wilk test. Normally distributed data were reported as mean (standard deviation, SD) and median (interquartile range, IQR). Non-normally distributed data were reported only as median (IQR). The difference between the two ratio variables was measured by the independent one-way t-test and two categorical variables by the chi-square or Fisher’s exact test.

Result

Study Characteristics

**Figure 1:**
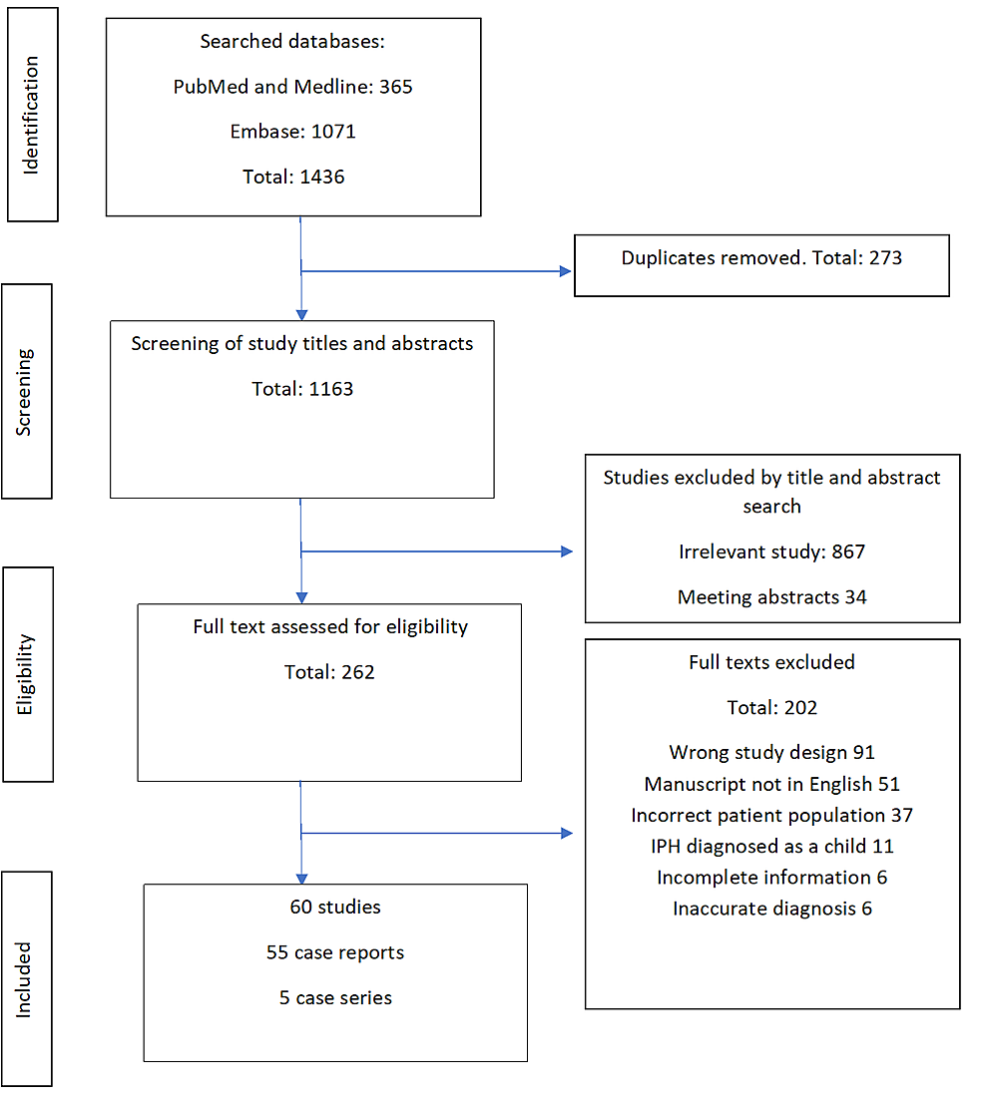
Flow-chart showing the selection of studies

A total of 60 studies that fulfilled the inclusion criteria were identified [2,11,14-69]. Figure 1 illustrates the study selection process. No prospective or retrospective studies were included for this review. We excluded a retrospective study consisting of nine patients, as the paper did not have adequate individual patient details [70]. Among the 60 studies, 55 papers were case reports that presented a single patient. The other five papers were small case series [30,36,40,64,71]. Patients were reported from all continents.

Subject Demographics

A total of 65 patients were included in this review. The patients were divided into two cohorts. Cohort A included patients with IPH without CD (49 patients). Cohort B consisted of patients with LHS and consisted of 16 patients [11,22,26-27,31,37,41,45,52-53,55-56,58-59,67,72].

The median and mean age of cohort A at the time of IPH diagnosis were 27 years (IQR, 27.5) and 35.49 years (SD, 16.9), respectively. The median and mean age of cohort B at IPH diagnosis were 27 years (IQR, 17) and 26.62 years (SD, 10.04), respectively. The difference in mean age at diagnosis did not reach statistical significance (independent sample one-way t-test, p=.051).

The median and mean age at symptom onset in cohort A were 27 years (IQR, 27) and 34.33 years (SD, 17.54), respectively. The median and mean age at symptom onset were 26 years (IQR, 11) and 25.53 years (SD, 10.33), respectively, in cohort B. The difference in mean age at symptom onset was statistically significant (independent sample one-way t-test, p=.012).

Twenty-eight out of 49 (57.1%) patients were males in cohort A compared to 12/16 (75%) patients in cohort B. A detailed breakdown of the demographic data based on gender is presented in Table 1.

**Table 1:** Summary of comparative data between cohorts A and B IPH, idiopathic pulmonary hemosiderosis; IQR, interquartile range; SD, standard deviation

Patient demographics	Cohort A (IPH without CD)		Cohort B (IPH with CD)		P-value
Age at IPH diagnosis in years	Mean (SD)	Median (IQR)	Mean (SD)	Median (IQR)	
All patients	35.4 9 (16.90)	27 (27.5)	26.62 (10.04)	27 (17)	P=.051
Male	38.46 (19.10)	32.5 (27.75)	27.5 (7.39)	27 (13)	
Female	31.46 (12.78)	27 (25)	36 (15.23)	37 (28)	
Age at symptom onset in years	Mean (SD)	Median (IQR)	Mean (SD)	Median (IQR)	
All patients	34.33 (17.54)	27 (27)	25.53 (10.33)	26 (11)	P=.012
Male	36.92 (19.36)	30 (29)	24.33 (9.04)	25.5 (11)	
Female	30.44 (14.03)	24.5 (27.5)	30.33 (15.94)	26 ()	
Delay in diagnosis in weeks	Median		Median		
All patients	52		52		
Male	52		52		
Female	69		92		
Classic triad at presentation - percent	74.5%		93.8%		
Immunosuppressive therapy - percent	80.4%		40%		
Recurrence	56.8%		28.6%		p=.086
Survival	88.7%		100%		p=.098
Gender - male	57.1%		75%		

Presenting Symptoms of IPH

The presenting symptoms varied between cohorts A and B as follows: hemoptysis (77.6% vs 93.8%), dyspnea (83.3% vs. 81.3%), anemia (87.5% vs. 100%), cough without hemoptysis (24.5% vs. 18.8%), and chest pain (6.1% vs. 12.5%). Systemic symptoms, such as fever, night sweats, chills, fatigue, weight loss, or loss of appetite, were present in 32.7% vs. 50% of patients. Respiratory failure was reported in 8/49 (16.3%) patients in cohort A. In contrast, no patient in cohort B suffered from respiratory failure (Fischer’s exact test, p=.102).

Delay in the Diagnosis of IPH

The median delay from onset of symptoms to diagnosis of IPH was 52 weeks for both cohorts A and B (non-normally distributed data). More detailed data are given in Table 1. The mean difference in the diagnostic delay between cohort B and A was 71.57 weeks, which was not statistically significant (p=.154).

Laboratory and Radiologic Findings

The mean hemoglobin on admission for cohorts A and B were 8.8 (SD, 2.59) and 7.43 (SD, 2.35) gm/dL, respectively. The mean difference was not statistically significant, p=.088. The median serum ferritin levels were 91.85 ng/ml (IQR,117.43) for cohort A and 40.78 ng/ml (IQR, 510.61) for cohort B (non-normal data).

The radiologic findings in cohorts A and B were as follows: bilateral infiltrate (91.5% vs.100%), emphysema or cystic changes (19.1% vs. 18.8%), and interstitial changes (27.7% vs. 25%). Pleural effusion was present in only one patient with LHS [67]. The classic triad was present in 74.5% vs. 93.8% of patients.

Autoantibody Testing and Type of Autoantibodies

Evaluation for at least one autoantibody was performed in 58/63 (92.1%) patients in the entire cohort. The completeness of antibody testing varied widely. Evaluation for CD-specific antibodies was performed in 22/63 (34.9%) patients. Seventeen out of 63 (26.9%) patients tested positive for an autoantibody. Thirteen out of 17 (76.4%) of patients tested positive for anti-CD antibodies. The prevalence of CD antibodies among tested patients was 13/22 (59%). The details of these patients with LHS are provided in Tables 2-3.

**Table 2:** The reported 16 adult cases diagnosed as idiopathic pulmonary hemosiderosis (IPH) having a workup for autoantibodies, including evidence of celiac disease (Lane-Hamilton syndrome) ANA, antinuclear antibody; ANCA, antineutrophil cytoplasmic antibody; c-ANCA, cytoplasmic ANCA; CD, celiac disease; dsDNA, double-stranded DNA; EF, ejection fraction; GBM, glomerular basement membrane; IPH, idiopathic pulmonary hemosiderosis; P-ANCA, perinuclear ANCA; RF, rheumatoid factor; RNP, ribonucleoprotein; TTG, tissue transglutaminase

Author	Age at IPH diagnosis (year)	Gender	Race/ County	Presenting symptoms	Duration of presenting symptoms	Age at respiratory symptom onset (year)	Delay in IPH diagnosis	Autoantibody tested	Positive antibody	Temporal relation with initial diagnosis	Prominent GI symptoms	Other organ involvement
Austin et al.2021 [67]	39	M	NS/USA	Left-sided chest pain and exertional dyspnea	1-year Similar presentation 1 year ago (had left-sided pleural effusion)	29 First episode of hemoptysis 10 years before presentation	At least 1 year	ANA, ANCA, anti-GBM: negative	Anti-TTG	CD diagnosed 5 years before IPH but the patient had hemoptysis 5 years before the diagnosis of CD	Minimal Workup for CD done for chronic anemia	Membranous nephropathy
Karatas et al.2016 [58]	21	M	NS/Turkey	Hemoptysis, respiratory difficulty, lethargy, and fatigue	2 days	21	None	ANA, ANCA, anti-dsDNA, anti-GBM: Negative	Anti-TTG Anti-gliadin antibody	At the time of IPH diagnosis	No	None
Popp et al. 2016 [59]	48	F	NS/Romania	Recent hemoptysis with severe acute hemoptysis	NS	NA	NA	ANA, ANCA, anti-dsDNA, anti-GBM, anti-Ro/La, anti RNP: negative	Anti-gliadin and endomysial antibody	At the time of IPH diagnosis	No	None
Berger et al.2015 [55]	26	F	NS/USA	Cough, hemoptysis, exertional shortness of breath, and fatigue	Cough and hemoptysis for 6 months. Fatigue and SOB for 2 months	26	None	ANA, ANCA, anti-dsDNA, anti-GBM: Negative	Anti-gliadin and endomysial antibody	At the time of IPH diagnosis	No	None
Khilnani et al.2015 [56]	19	M	NS/India	Hemoptysis, dyspnea, and fatigue	3 years	16	3 years	ANA, ANCA, RF, anti-cardiolipin, anti-GBM: Negative	Anti-TTG antibody	At the time of IPH diagnosis	No	Dilated cardiomyopathy, EF 25%
Dos Santos et al.2012 [72]	29	M	White/Brazil	Cough, shortness of breath, weight loss	5 months	29	None	ANA, ANCA, P-ANCA, C-ANCA, anti-GBM: Negative	Anti-gliadin and endomysial antibody	At the time of IPH diagnosis	No	None
Patrucco et al.2012 [52]	27	M	NS/Italy	Recurrent hemoptysis at age 27. Worsening dyspnea at 31	Admission to the hospital with anemia, duodenal hemorrhagic telangiectasia at age 22 (no chest X-ray abnormalities)	27	None	Negative IgA anti-TTG and anti-endomysial antibody at age 27	Positive anti-gliadin, endomysial, and TTG antibody at 31	4 years after diagnosis of IPH	No	Myocarditis, atrial and ventricular tachyarrhythmia at age 29. Dilated cardiomyopathy (EF 24%) at age 31. EF 57% at age 35
Singhal et al.2013 [53]	27	M	NS/India	Hemoptysis with up to 400ml of fresh blood	2 months	27	None	ANA, ANCA, P-ANCA, C-ANCA, anti-GBM: Negative	Anti-IgA TTG	At the time of IPH diagnosis	No	None
Nishino et al.2010 [45]	50	F	NS/USA	Hemoptysis, shortness of breath, dizziness, and orthostasis				ANCA, P-ANCA, C-ANCA, anti-GBM, and anti-dsDNA: Negative	ANA 1:160 Anti-TTG and endomysial antibody	Patient had a history of CD, diagnosed 10 years ago	Not at the time of DAH	None
Mayes et al.2008 [41]	40	M	Hispanic/USA	Hemoptysis and shortness of breath	3 months	40	None	ANCA, P-ANCA, C-ANCA, anti-GBM, and RF: Negative	Anti-gliadin antibody	At the time of IPH diagnosis	No	None
Jecko et al.2007 [37]	20	F	NS/UK	Hemoptysis, chest pain, fever	3 years	17	None	ANCA, anti-GBM: negative	None	Diagnosed with CD 12 years ago	NS	None
Malhotra et al.2004 [31]	28	M	NS/India	Hemoptysis, worsening dyspnea	5 days	24	4 years	ANA, ANCA, anti-GBM: negative	Anti-IgA endomysial antibody	At the time of IPH diagnosis	No	None
Bouros et al.1994 [27]	19	M	NS/Greece	Recurrent hemoptysis	More than a year	18	1 year	ANCA, anti-GBM, RF, anti-Scl-70, and anti-dsDNA: Negative	Anti-reticulin and gliadin antibodies	At the time of IPH diagnosis	No	None
Pacheco et al.1991 [26]	22	M	NS/Spain	Hemoptysis, exertional shortness of breath, and arthralgia	15 years	7	15 years	ANA, anti-GBM, RF: negative	Anti-reticulin antibody 1:640	At the time of diagnosis of IPH	No	None
Ludmerer et al.1986 [22]	36	M	NS/USA	Recurrent pneumonia: cough and hemoptysis	2 months	36	None	ANA, RF: negative	NA	2 months after IPH diagnosis	Yes	None
Lane et al.1971 [11]	23	M	NS/England	Hemoptysis at age 18	NS	NS	None	RF: negative	NA	At the time of IPH diagnosis	Yes, since age 9	None

**Table 3:** Treatment and outcomes of the 16 adult cases with IPH and celiac disease (Lane-Hamilton syndrome) BAL, bronchoalveolar lavage; CD, celiac disease; EF, ejection fraction; EM, electron microscopy; GFD, gluten-free diet; GGO, ground-glass opacity; GI, gastrointestinal; HLM, hemosiderin-laden macrophages; IPH, idiopathic pulmonary hemosiderosis; LLL, left lower lobe; NS, not specified; PFT, pulmonary function tests; VATS, video-assisted thoracoscopic surgery

Author	Modality of IPH diagnosis	Chest radiology	Bronchoscopy/Gastric aspiration findings	Lung histopathology	Small intestinal histopathology	Serum ferritin	Initial treatment	Recurrence of IPH	Clinical course of CD	Follow-up (years)	Respiratory outcomes
Austin et al.2021 [67]	VATS	Emphysema, interstitial infiltrate, mediastinal adenopathy, right lower lobe consolidation, and right-sided pleural effusion	NA	Intraalveolar HLM, type 2 pneumocyte hyperplasia	Biopsy comparable to CD but not specified	Elevated	GFD started 1 year before the index admission with questionable adherence. During index admission, patient discharged on CS and GFD	Yes. NS if there was recurrence with CS and GFD	NS	NS	NS
Karatas et al.2016 [58]	BAL	Bilateral GGO	Mildly hemorrhagic fluid on BAL Hemosiderin-laden macrophages	Not performed	Duodenal biopsy: Intraepithelial plasma cells and eosinophils, Villous atrophy, and crypt hyperplasia	Low normal	High dose corticosteroid with excellent response. The patient was discharged on GFD once CD was diagnosed	None	Hemoglobin normalized	0.67 years	Completely normal
Popp et al.2016 [59]	BAL	Diffuse pulmonary sclero-emphysema suggestive of alveolar hemorrhage	No malignancy, mucus, rare RBS, and HLM on BAL	Not performed	Nodular duodenal bulb Duodenal biopsy: Villous atrophy, crypt hyperplasia, and increased intraepithelial lymphocytes	NS	GFD and tapering steroid	None	Hemoglobin normalized	0.5 years	Clinical and radiologic improvement
Berger et al.2015 [55]	BAL and transbronchial biopsy	Bilateral GGO	Normal bronchial mucosa	Conglomeration of HLM in the alveolus and thickening of alveolar interstitium, no vasculitis, or inflammatory infiltrate	Jejunal biopsy: consistent with CD	NS	CS and GFD. CS was tapered off after 9 months of therapy	None	Normal respiratory function and normal hemoglobin	4 years	Normal respiratory function Mild subpleural residual fibrosis
Khilnani et al.2015 [56]	BAL and transbronchial biopsy	Bilateral GGO	NS	Type 2 pneumocyte hyperplasia, HLM in the alveolar space, and chronic inflammatory cells	Duodenal biopsy: subtotal villous atrophy and crypt hyperplasia (Marsh IIIb)	low	GFD	None	Improved hemoglobin	2 years	Remission of hemoptysis, improved PFT EF improved to 35%
Dos Santos et al.2012 [72]	BAL	Bilateral GGO and consolidation	HLM from BAL	Not performed	Duodenal biopsy: Villous flattening, intraepithelial lymphocyte	NS Iron deficiency anemia	GFD	None	Normalization of hemoglobin	0.5 years	Completely normal respiratory function
Patrucco et al.2012 [52]	BAL and transbronchial biopsy at age 29	Bilateral reticulonodular infiltrate	NS	HLM in the alveolus without any evidence of vasculitis	Duodenal biopsy at 31 consistent with CD. The duodenal biopsy at 22 showed nonspecific findings (sample was not available for re-examination)	Normal	CS with tapering dose at age 27	Yes. Readmission to the hospital at 31 for worsening dyspnea. Started on high-dose CS, AZA, and the GFD. Immunosuppression tapered off in 3 months	Normalization of hemoglobin	4 years	Normalization of PFT
Singhal et al.2013 [53]	BAL	Bilateral diffuse alveolar infiltrate in the mid and lower lung zone	Numerous HLM	Not performed	Duodenal biopsy: Partial villous atrophy, increased intraepithelial lymphocyte and plasma cells in lamina propria	NS Iron deficiency anemia	GFD	None	Normalization of hemoglobin, weight gain	1 year	Completely normal pulmonary function
Nishino et al.2010 [45]	Bronchoscopy and lung biopsy	Bilateral GGO (more prominent in LLL and right upper and middle lobe)	NS Bronchoscopy consistent with DAH	Intraalveolar HLM	NS (patient was diagnosed with CD 10 years ago)	NS Iron deficiency anemia	NS	NS	NS	NS	NS
Mayes et al.2008 [41]	Bronchoscopy and VATS lung biopsy	Bullous disease in bilateral lower lobes. Interstitial opacity in upper lobes	Bronchoscopy not diagnostic	Chronic alveolar hemorrhage, interstitial hemosiderin deposition, hemosiderin deposition in the elastic fibers of the interstitium and vessel wall	Duodenal biopsy: Villous blunting, increased intraepithelial lymphocyte, and plasmacytosis in lamina propria	NS iron deficiency	GFD	Yes CS started after PFT declined after 2 months. Later, AZA was added as myopathy developed with high steroid	NS	NS	No more hemoptysis
Jecko et al. 2007 [37]	BAL	Diffuse bilateral infiltrate	HLM from BAL	Not performed	Small bowel biopsy: subtotal villous atrophy and lymphocytic infiltrate	NS. Iron deficiency anemia	GFD (not compliant)	Yes. No immunosuppressive therapy	Ongoing anemia	2 years	Persistent hemoptysis, approximately 2 times a month
Malhotra et al.2004 [31]	BAL and transbronchial biopsy	Diffuse GGO in bilateral mid and lower lung zones	Hemorrhagic fluid with >80% HLM	Intraalveolar hemorrhage, interlobular septal thickening. No capillaritis, granulomatosis, or necrosis. No immunocomplex deposition. EM showed no basement membrane abnormalities	Duodenal biopsy: Villous atrophy, intraepithelial lymphocytes, and plasma cells in the lamina propria	NS Microcytic hypochromic anemia	GFD	None	Improved hemoglobin and reduced antibody titer	0.5 years	No recurrence of hemoptysis
Bouros et al.1994 [27]	BAL and transbronchial biopsy	Diffuse bilateral alveolar infiltrate primarily in the lower lobes	Progressive bloody return on BAL HLM	Nonspecific alveolar hemorrhage, mild to moderate thickening of alveolar septa, HLM. No vasculitis, granulomatosis, necrosis, or immunocomplex deposition	Jejunal biopsy: Villous atrophy	NS Iron deficiency	GFD	NS	NS	0.5 years	Continued clinical improvement
Pacheco et al.1991 [26]	BAL and transbronchial lung biopsy	Bilateral micronodular opacity	HLM on BAL	EM showed focal rupture of the basement membrane. No vasculitis, granuloma, or necrosis	Jejunal biopsy: Subtotal villous atrophy	NS. Iron deficiency anemia	GFD	None	The antibody disappeared and jejunal biopsy was normal	0.5 years	No recurrence of hemoptysis
Ludmerer et al. 1986 [22]	Lung biopsy	NA	NA	Intraalveolar bleed, HLM, hemosiderin deposition in the interstitium, type 2 alveolar cell hyperplasia. No immunocomplex deposition. No abnormalities of basement membrane on EM	Small bowel biopsy: Total villus atrophy. Intraepithelial lymphocyte and crypt hyperplasia	NS Iron deficiency	GFD	NS	The GI symptoms improved	NS	Stabilization of pulmonary function
Lane et al.1971 [11]	Sputum and lung biopsy	Bilateral patchy infiltrate in lower lobes	Iron laden macrophages in sputum	Lung biopsy consistent with IPH	Jejunal biopsy: subtotal villous atrophy	NS Iron deficiency	Azathioprine	Yes	Persistent GI symptoms	At least a year	Ongoing small volume hemoptysis

Diagnosis of CD and Associated Autoantibodies

The prevalence of CD in patients with IPH in the predefined period was 16/64 (25%). All but one study specified histopathologic analysis of small bowel biopsy to confirm CD [45]. Three out of 16 patients were known to have CD before the diagnosis of IPH [37,45,67]. Two patients were diagnosed with CD after IPH was identified [22,52]. In the remaining 11 patients, the diagnosis of IPH and CD were made simultaneously. Ten of these 11 patients initially tested positive for anti-CD antibody, followed by a biopsy. Only two of 15 (13.3%) patients reported any noticeable gastrointestinal symptoms. Workup for CD was performed either because of the known association with IPH or to evaluate for disproportionate anemia.

Regarding specific anti-CD antibodies, the positivity was as follows: TTG, 7/12, endomysial antibody 6/8, anti-gliadin antibody 6/10, and anti-reticulin antibody 2/2. Three studies did not report any serologic workup for CD, and the diagnosis was made by small bowel biopsy [11,22,37].

Diagnosis of IPH

A definitive diagnosis of IPH was made by lung biopsy in 41/49 (83.7%) patients in cohort A and 11/16 (68.8%) patients in cohort B. The biopsy techniques employed between cohorts A and B are as follows: surgical lung biopsy (46.3% vs. 27.2%), video-assisted thoracoscopic surgery (21.95% vs. 18.18%), and transbronchial lung biopsy (TBLB; 24.39% vs. 54.54%). One of the TBLB was a cryobiopsy [67]. In cohort A, the histopathologic analysis was performed post-mortem in three (7.31%). Out of eight patients in cohort A who did not have lung histopathology, five were diagnosed by the demonstration of HLM from bronchoalveolar lavage (BAL), three by a progressively bloody return from BAL, and one by HLM in sputum. All five patients in cohort B without lung biopsy were diagnosed by HLM from BAL.

Treatment

Following the diagnosis of IPH, immunosuppressive medications were used in 37/46 (80.4%) patients in cohort A. Corticosteroid (CS) was the most popular first-line medication 35/37 (94.6%). The other two patients were started on azathioprine (AZA) as first line therapy [17,20]. Ten out of 35 (28.6%) patients who initially received CS required a second-line medication. Seven out of 10 received AZA, 2/10 were given antimalarials, and one patient was treated with cyclophosphamide (CYC). In contrast, only 6/15 (40%) of patients in cohort B received immunosuppression. Five of these patients received CS, and one was treated with AZA. All patients except one were treated with GFD [11]. The treatment for CD was unknown at the time of the original report by Drs. Lane and Hamilton [11]. As a result, this patient received therapy with AZA. The treatment was not specified in one patient [45]. Nine out of 15 (60%) patients were discharged only on GFD without corticosteroid (CS) therapy [22,26-27,31,37,41,53,58,72]. Only one of these patients subsequently required CS due to declining pulmonary function tests [41]. Three patients were initially treated with GFD and CS, and successfully tapered off steroids [52,55,59]. One patient, in addition to GFD, required long-term CS [67]. However, the compliance to GFD in this patient was questionable.

Clinical Course and Follow-Up

The median follow-up period in cohorts A and B were 1.4 (IQR, 3.21) vs. 0.75 (IQR, 2) years, respectively (non-normally distributed data). The mean difference between the cohorts was not statistically significant (p=.149). Recurrence during follow-up was reported in 56.8% (21/37) of patients in cohort A and 28.6% (4/14) of patients in cohort B (chi-square test, p=.086). Two of these patients were not on a gluten-free diet (GFD) at the time of recurrence [11,52]. The other two patients still had IPH symptoms despite being on GFD [41,67]. Ten out of 47 (21.3%) patients died in cohort A, but all patients were alive at the time of the follow-up in cohort B (Fisher’s exact test, p=.098).

Discussion

Since first described in 1971 by Drs. Lane and Hamilton [11], multiple papers have been published reporting the coexistence of IPH and CD in adult patients. However, a comparative analysis between patients with IPH and LHS has never been done to the best of our knowledge. Therefore, we have performed a systematic review of adult patients with IPH with or without concomitant CD in the past 50 years and reported the similarities and differences between these two groups of patients.

Our study reaffirmed the previous notion that IPH is more common among males in the adult population, but unlike the study by Chen et al. [73], most of our patients (in both cohorts) were diagnosed before the age of 30 years. The patients with LHS are younger at symptom onset (statistically significant) and at the time of diagnosis of IPH compared to patients without CD, findings that had not been reported before.

CD or gluten-sensitive enteropathy is a common autoimmune disease with an adult population prevalence of approximately 1% [74]. CD is more prevalent in the pediatric population and in women than men [74]. Although LHS has been a known entity for half a century, the prevalence of LHS among IPH patients has been unknown. In children with IPH, the prevalence of anti-CD antibodies was 25.9% [13]. Based on this review, the prevalence among adults is also 25%. However, when only patients who had been specifically evaluated for CD by serologic testing are considered, the prevalence is as high as 59%. The fact that the prevalence is so high in children and adults, and the number is significantly higher than the general population, raises concerns for unifying the underlying pathobiology of this co-occurrence. Several hypothetical mechanisms of DAH in CD have been considered, such as food allergen-induced immune-complex deposition, anti-reticulin antibody-mediated alveolar basement membrane damage, and molecular mimicry of an infectious pathogen [75-77]. However, there has never been any histopathologic proof to support any of them.

The pathobiology of DAH in IPH is currently unknown. Genetic, environmental, and allergic hypotheses have been proposed but do not appear to be of much merit [1]. The immunologic hypothesis is the most popular among clinicians. We have proposed a new pathogenetic hypothesis for the occurrence of DAH in IPH [78]. We believe that the etiology of DAH is cytokines and chemokines, such as histamine, eosinophilic cationic protein, and vascular endothelial growth factor-induced changes in pulmonary capillaries that promote increased gap between endothelial cells leading to RBC extravasation in the alveolar space without any structural vascular damage. The cytokines are expressed by the immunologic cells, such as eosinophils, basophils, and lymphocytes, when exposure to an unknown antigen occurs locally in the lung or through another route such as enterally. Moreover, an overall immune dysregulation in IPH could explain the susceptibility among these individuals to develop autoantibodies and other immune-mediated diseases [16,79-83]. We have proposed a new name, immune-mediated pulmonary hemosiderosis (ImPH), to focus on the immunologic causation of DAH [84-85].

The presenting symptoms in IPH are nonspecific. Patients generally present with hemoptysis of variable severity ranging from intermittent episodes of blood-streaked sputum to life-threatening pulmonary hemorrhage [38,64]. Other symptoms include cough with or without sputum production, dyspnea, and chest pain. Although hemoptysis is common in adult patients, pediatric patients may not have hemoptysis due to inadvertent swallowing of the sputum. Systemic symptoms affect approximately half of the patients [86]. The classic triad consists of hemoptysis, radiologic chest abnormalities, and anemia. Anemia appears to be more prevalent in LHS patients than patients without CD. All patients with LHS in our study suffered from anemia. This could be due to chronic malabsorption and resultant iron deficiency in addition to chronic alveolar hemorrhage. The most common radiologic findings during the acute phase of the disease include bilateral ground-glass opacities on chest computed tomography [87]. In addition, areas of consolidation may also be present [3]. Typically, the mid and lower lung zones are affected more than the upper zones, but this is not universal [1]. Other less common radiologic changes, such as emphysema, cystic changes, and interstitial infiltrate, were seen in similar frequencies between IPH and LHS patients [88]. The median delay in the diagnosis from symptom onset was one year and similar between both groups.

Although IPH is considered an immunologic disease [84], unlike other autoimmune diseases, multisystem involvement by IPH has never been definitively proven. There are reports of myocardial dysfunction [89], myocarditis [14], conduction system [90] and segmental wall motion abnormalities [15], and sudden death [91] in patients with IPH, but histopathologic analysis of myocardial tissue has not demonstrated hemosiderin deposition as the underlying etiology [14,89-90]. We have identified two patients in cohort B who also suffered from dilated cardiomyopathy [52,56]. Previous studies have demonstrated a higher prevalence of anti-CD antibodies in patients with autoimmune myocarditis [92-93]. Whether IPH, CD, and autoimmune myocarditis stem from the same immunologic process is unknown at this time. Genome-wide association studies may shed more light on this association. One patient in cohort B also suffered from membranous nephropathy [67].

One of the crucial and interesting findings in this study was the lack of GI symptoms in patients with LHS [26-27,31,37,41,45,52-53,55,56,58-59,67,72]. Less than 20% of patients complained of any significant GI symptoms at the time of IPH diagnosis. Similar findings were also seen in pediatric patients with LHS [77]. Although malabsorption leading to developmental delay is common in children with LHS, this is rarely the case in adults [53]. As a result, reliance on clinical symptoms only may underestimate the possibility of CD in IPH patients, and patients should undergo workup for CD. The workup for CD often starts with serologic assays for anti-CD antibodies. These antibodies are very sensitive and specific for CD. The commonly tested antibodies are anti-tissue transglutaminase (anti-TTG) and anti-endomysial IgA antibodies [94]. In patients with selective IgA deficiency, IgG antibodies against these antigens could be tested. Other autoantibodies, such as anti-gliadin and ant-reticulin antibodies, have fallen out of favor. Positive antibody testing is generally followed by a small intestinal biopsy for histopathologic proof of CD. The typical histopathologic findings in CD are intraepithelial lymphocytosis, infiltration of the lamina propria by plasma cells, neutrophils and eosinophils, crypt hyperplasia, and variable degrees of villous blunting [95]. To standardize histopathologic reporting, pathologists often use a modified Marsh classification. According to this classification, the severity of small bowel involvement ranges from stage I to stage IIIc, where stage I represents the earliest changes characterized by only increased intraepithelial lymphocyte count (>40 cells/100 enterocytes), and stage IIIc demonstrates total villous atrophy and crypt hyperplasia in addition to IEL [96]. The histopathologic terminologies varied among authors in the included manuscripts, likely due to regional differences in reporting practice.

The definitive diagnosis of IPH requires histopathologic analysis of the lung parenchyma. However, this practice does not appear to be universal, and identification of HLM from BAL or even sputum has been considered adequate by authors in the previous reports [21]. As HLM can be present from BAL even without DAH (bleeding from the airways will accumulate in the alveolus and subsequently HLM can be present after 48-72 hours), it is important to make a diagnosis of DAH by demonstrating progressively bloody fluid return on serial BAL [2,97]. Transbronchial biopsies can be easily performed during bronchoscopy, but the samples are of small size. Transbronchial cryobiopsy provides larger and better-preserved samples with a higher risk of bleeding complications [63,98]. The surgical lung biopsy, either by thoracotomy or video-assisted thoracoscopic surgery (VATS), was performed in 68% of patients in the IPH and 45% of the LHS cohort. It is possible that due to the previously known association between CD and IPH, clinicians were more comfortable making a diagnosis of IPH from BAL and transbronchial biopsy (TBB) without obtaining a surgical lung biopsy, which is more invasive.

Systemic CS represents the first-line therapy during active disease as well as for maintenance therapy [99]. Approximately one-third of reported adult patients have required a second-line immunosuppressive agent to control their disease [86]. Unconventional therapies, such as liposteroid, leflunomide, and mesenchymal stem cell transplant, have also been tried [100-102]. In contrast, GFD alone was effective in controlling DAH in the majority of patients with LHS. Only one patient in the literature required additional immunosuppressive therapy for DAH while following GFD. Patients who were simultaneously started on CS and GFD were able to come off of all immunosuppressive therapy within a short period of time. Therefore, unless the patient suffers from life-threatening alveolar hemorrhage, most patients can be started on GFD prior to initiation of corticosteroid (CS). If CS is started, care should be taken to rapidly taper off and discontinue such therapy as it is associated with a significant side-effect profile.

Even during the short follow-up period, recurrence was common among the entire cohort. A trend towards less recurrence was noted in the LHS cohort, especially when the patient was compliant with GFD. Similarly, survival was also better in that cohort. No patient with LHS died during the follow-up. These findings have raised the possibility of whether IPH in adults has different endotypes and phenotypes. Patients with LHS may have a milder form of the disease with less recurrence risk and better survival compared to IPH patients without CD. If that were true, evaluation for CD is crucial as the treatment intensity with immunosuppressive therapy can be modified based on the presence or absence of CD. As two patients in our study developed CD antibodies after the diagnosis of IPH, and it may be prudent to intermittently check for them during follow-up [22,52].

Strength and limitations of the study

Our study has several limitations. First, the number of patients with LHS is modest, likely due to the rarity of the disease. Second, most patients have been reported as ‘case reports’ and therefore may not have included all pertinent parameters. Third, we excluded literature published in a non-English language, which may have reduced the number of cases. Fourth, the follow-up period was limited, and the true prognosis may not have been clearly evident fifth, there was a risk of bias in the reporting, as only selected cases with particularly positive or negative outcomes may have been reported. Despite that, this study adds significantly to the existing literature on adult IPH. The similarities and discrepancies between IPH and LHS regarding patient demographics, clinical presentation, treatment, and prognosis have never been studied before.

## Conclusions

IPH is a rare disease of unknown etiology and pathogenesis. In this manuscript, we have reported the similarities and differences between adult patients with IPH and LHS. From our review of the literature, we have identified a high prevalence of CD among adult patients with IPH. Patients with LHS are younger and start experiencing respiratory symptoms earlier than adult IPH patients. The classic triad is more common in LHS, but the delay in diagnosis between both groups is similar. Fewer patients with LHS undergo lung biopsy for a diagnosis of IPH than patients without CD. GFD is often the first line of treatment and appears to be efficacious. Some patients require additional immunosuppressants and, more often than not, are able to come off if it. The risk of recurrence is likely lower, and the chance of survival is higher among patients with LHS than IPH patients. A new diagnosis of IPH in adults should prompt serologic evaluation for CD.
